# Body Composition Changes in Hospitalized Patients with Community-Acquired Pneumonia

**DOI:** 10.3390/jcm14155460

**Published:** 2025-08-03

**Authors:** Ryuji Sugiya, Osamu Nishiyama, Masashi Shiraishi, Kazuya Yoshikawa, Kyuya Gose, Ryo Yamazaki, Takashi Oomori, Akiko Sano, Shinichi Arizono, Yasushi Uchiyama, Yuji Higashimoto, Hisako Matsumoto

**Affiliations:** 1 Department of Integrated Health Sciences, Nagoya University Graduate School of Medicine, Nagoya 4618673, Japan; sugiya.ryuji.r7@f.mail.nagoya-u.ac.jp (R.S.); uchiyama.yasushi.f9@f.mail.nagoya-u.ac.jp (Y.U.); 2 Department of Respiratory Medicine and Allergology, Kindai University Faculty of Medicine, Osakasayama 5898511, Japan; k-yoshikawa@med.kindai.ac.jp (K.Y.); gk042012@med.kindai.ac.jp (K.G.); ryo-y@med.kindai.ac.jp (R.Y.); t-oomori@med.kindai.ac.jp (T.O.); sanoa@med.kindai.ac.jp (A.S.); 3 Department of Rehabilitation Medicine, Kindai University Hospital, Osakasayama 5898511, Japan; masashi-shiraishi@med.kindai.ac.jp; 4 School of Rehabilitation Science, Seirei Christopher University, Hamamatsu 4338558, Japan; shinichi-a@seirei.ac.jp; 5 Department of Rehabilitation Medicine, Kindai University Faculty of Medicine, Osakasayama 5898511, Japan; yhigashi@med.kindai.ac.jp

**Keywords:** body composition, community-acquired pneumonia, fat mass, hospitalization, skeletal muscle

## Abstract

**Background**: The influence of hospitalization owing to pneumonia on changes in body composition has not been specifically reported. We conducted a prospective cohort study of patients with community-acquired pneumonia (CAP) requiring hospitalization to test the hypothesis that hospitalization affects body composition. **Methods**: Sixty-four consecutive patients with CAP were recruited. Body composition was measured within 24 h of admission and 24 h before discharge using bioelectrical impedance analysis. The association between changes in body composition and variables obtained at admission was investigated. Index values were calculated as weight divided by height squared. **Results**: The mean age of the patients was 76.0 ± 8.7 years (78.1% males). The median length of hospitalization was 12.0 days. Weight, body mass index (BMI), skeletal muscle (SM), SM index, fat-free mass (FFM), and FFM index significantly decreased (*p* < 0.001 for each), but fat mass (FM) and FM index did not. The serum total protein level was the only independent predictor of the lowest quartile of change in SM index (<−0.4) after adjusting for age and sex (*p* = 0.004). **Conclusions**: In summary, weight and BMI significantly decreased during hospitalization in patients with CAP, which was attributed to SM reduction. Patients with low serum total protein levels on admission were at risk of an accelerated decrease in the SM index. Nutritional intervention and rehabilitation are important for these patients.

## 1. Introduction

Pneumonia is a common and serious disease. Despite substantial progress in therapeutic options, pneumonia remains a major cause of morbidity. Even if patients survive pneumonia, hospitalization due to pneumonia may increase the risk of sarcopenia and recurrence of the disease. In older patients with community-acquired pneumonia (CAP), low muscle mass is associated with increased long-term mortality [1]. Similarly, in older patients with aspiration pneumonia, loss of muscle mass is a potential risk factor for 90-day mortality [2]. In 2021, pneumonia was the fourth leading cause of death in Japan, a super-aged society [3]. As populations age in developed countries, pneumonia management, including the prevention of sarcopenia, will become increasingly important. However, the effect of CAP on skeletal muscle (SM) loss during hospitalization remains unclear [4,5]. In particular, no evidence of changes in body composition has been reported.

Pneumonia is known as an inflammatory disease. Inflammation is a key driver of muscle protein loss [6]. Interleukin (IL)-6, a representative pro-inflammatory cytokine, can directly induce SM atrophy in healthy rat models [7], suggesting that pneumonia accompanied by severe inflammation may influence muscle wasting. The molecular mechanism of muscle atrophy induced by inflammation can also be explained by the activation of calpains and caspase-3, as well as autophagy [8]. In addition, baseline patient characteristics, such as nutritional status, may influence SM loss during hospitalization. Therefore, we conducted a prospective cohort study of patients with CAP who required hospitalization to test the hypothesis that hospitalization affects body composition and to clarify the factors that may be associated with the outcome.

## 2. Materials and Methods

### 2.1. Patients’ Population

This was a single-center, prospective cohort study of patients with CAP who were hospitalized in the general wards at our university hospital. Only adults aged ≥18 years were eligible. Sixty-four consecutive patients with CAP were recruited between March 2019 and May 2021. The patients received standard treatment for CAP during hospitalization in accordance with the Japanese Respiratory Society guidelines for the management of pneumonia in adults [3]. Variables, including blood and biochemical data and body composition, were evaluated within 24 h of admission and discharge. Patients who could not stand by themselves and/or those who had metallic implants in their bodies were excluded, as body composition could not be assessed using a bioelectrical impedance analysis (BIA) device. Patients with a history of stroke and/or neuromuscular disease were also excluded because their handgrip strength and tongue pressure were impaired. Patients with missing anterior teeth were also excluded, as they could not bite down on the equipment used to measure tongue pressure. The study protocol was approved by the Ethics Committee of the Kindai University Faculty of Medicine (No. 30-200). Written informed consent was obtained from all study participants. The study was conducted in accordance with the relevant guidelines and regulations of the Declaration of Helsinki.

### 2.2. Pneumonia Severity

Pneumonia severity was graded according to the A-DROP and CURB-65 scoring systems [9,10]. The A-DROP scoring system assesses the following parameters: age (≥70 years in male and ≥75 years in female patients), dehydration or blood urea nitrogen ≥ 21 mg/dL, oxygen saturation < 90%, disturbance in orientation (confusion), and systolic blood pressure ≤ 90 mmHg. The CURB-65 scoring system assesses the following parameters: confusion, blood urea nitrogen ≥ 20 mg/dL, respiratory rate ≥ 30/min, low blood pressure (systolic blood pressure < 90 mmHg or diastolic blood pressure ≤ 60 mmHg), and age ≥ 65 years. Each scoring system is composed of a 6-point scale (0–5). Physical status was measured using the Eastern Cooperative Oncology Group (ECOG) Performance Status Scale [11]. The oral feeding function was assessed using the Functional Oral Intake Scale [12].

### 2.3. Analysis of Body Composition

Body composition was measured using the InBody720 multi-frequency BIA method (InBody Japan Inc., Tokyo, Japan), a validated method for detecting respiratory diseases [13,14]. It was measured within 24 h after admission and within 24 h before discharge. Body weight, SM, fat-free mass (FFM), fat mass (FM), and other body composition variables were automatically recorded. The body mass index (BMI), SM index, FFM index, and FM index were calculated as weight divided by height squared. The extracellular water/total body water ratio, an indicator of body fluid overload, was also measured. The body composition was evaluated within 24 h of admission and 24 h before discharge. The measurements were taken between 4 p.m. and 6 p.m. just before dinner. Patients were encouraged to urinate and avoid excessive fluid intake prior to the measurement.

### 2.4. Evaluation of Handgrip and Tongue Strength

Handgrip strength was measured three times for both hands using a grip strength dynamometer (GRIP-D; Takei Scientific Instruments Co., Ltd., Niigata, Japan), and the mean value was used for evaluation. Maximum tongue pressure was measured using a JMS tongue pressure measuring device^®^ (JMS Co., Ltd., Hiroshima, Japan), a balloon-based tongue pressure measurement device according to the method, which was previously proposed [15]. Briefly, the probe was inflated to a baseline pressure of 19.6 kPa. To measure maximum tongue pressure, the balloon was positioned on the anterior palate with the lips closed. The patients raised their tongues and compressed the balloon onto the palate with maximal voluntary muscular effort for approximately 7 s. Measurements were taken three times at 1 min intervals, and the maximum value was recorded as the maximum tongue pressure. Handgrip and tongue strength were also evaluated within 24 h of admission and within 24 h before discharge.

### 2.5. Statistical Analysis

Values are shown as the mean ± standard deviation or the median with inter-quartile range. Comparisons of each measured value between on admission and before discharge were performed using the Wilcoxon signed-rank test. Differences in variables between the two groups were analyzed using the Mann–Whitney U test. Correlations between changes in variables and baseline factors were assessed using Spearman correlation analysis. Multivariate logistic regression analysis was also performed. Statistical significance was set at *p* < 0.05. All analyses were performed using SPSS 24.0 (IBM Corp., Armonk, NY, USA).

## 3. Results

### 3.1. Baseline Characteristics and Their Associations with Disease Severity

**Figure 1:**
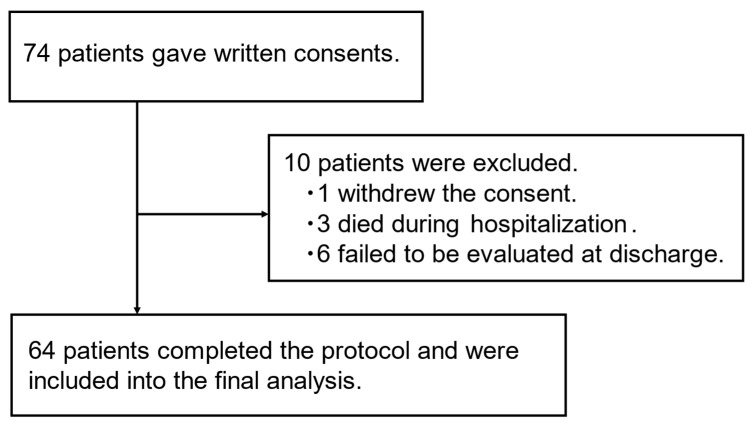
Flowchart of patient inclusion.

The study inclusion flowchart is shown in Figure 1. Eventually, 64 patients with CAP who were hospitalized for treatment were consecutively included in the analysis, although patient registration was suspended for a period owing to the outbreak of coronavirus disease 2019 (COVID-19). The patient characteristics at admission are summarized in Table 1. The mean age of the patients was 76.0 ± 8.7 years. The ages of our study population ranged from 58 to 89 years. Fifty (78.1%) of the sixty-four patients were male. Forty-two (65.6%) patients had lung comorbidities, such as chronic obstructive pulmonary disease and interstitial lung disease.

The median length of hospitalization and treatment with antibiotics were 12.0 (11.0–17.2) and 11.0 (9.0–15.3) days, respectively. The shortest duration of hospitalization was 5 days, while the longest was 34 days. The median A-DROP and CURB-65 scores were 1, indicating that most patients had mild-to-moderate disease severity. Regarding the association between body composition at admission and pneumonia severity, the CURB-65 score was negatively associated with FM (ρ = −0.32, *p* = 0.01), FM index (ρ = −0.27, *p* = 0.03), and BMI (ρ = −0.34, *p* = 0.01) at admission; however, the A-DROP score was not associated with any body composition parameters.

### 3.2. Changes in Variables from Admission to Discharge

**Figure 2:**
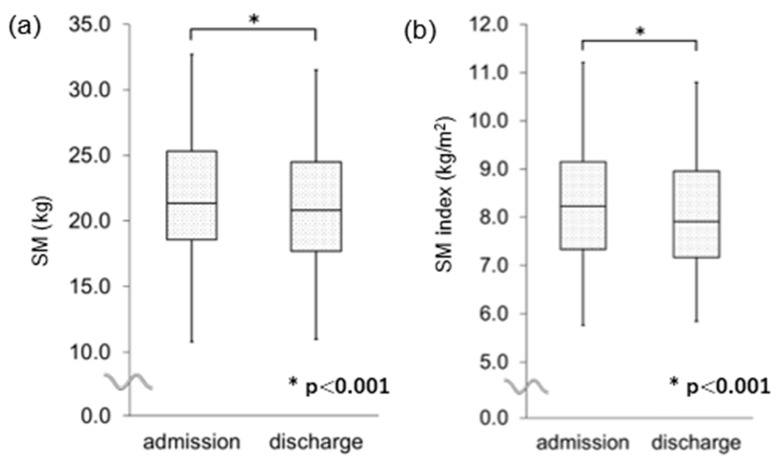
Comparison between variables of skeletal muscle at admission and at discharge: (**a**) SM and (**b**) SM index, respectively. SM, skeletal muscle; FM, fat mass. Δ = value at discharge minus value at admission. The box represents the median value and interquartile range, while the whiskers indicate the full spread of the data.

The changes in the variables from admission to discharge are summarized in Table 2. Whole blood cell count, serum C-reactive protein (CRP), total protein, ECOG performance status, body weight, and BMI significantly decreased. Regarding values related to body composition, SM, SM index (Figure 2), FFM, and FFM index significantly decreased; however, values related to FM, FM index, and extracellular water/total body water did not. While handgrip strength in the dominant hand, maximal tongue pressure, and Functional Oral Intake Scale score did not change during hospitalization, handgrip strength in the non-dominant hand significantly decreased. There were no differences in changes in variables between patients with and without lung comorbidities.

### 3.3. Characteristics of Patients with Accelerated Decrease in Body Composition

**Figure 3:**
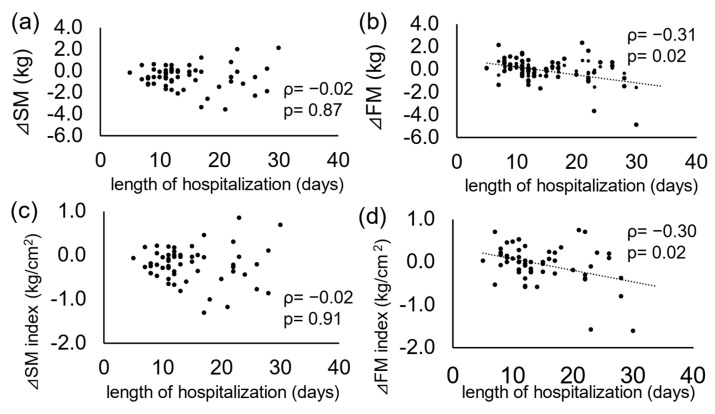
The relationship between changes in variables of the body composition and length of hospitalization: (**a**) SM, (**b**) FM, (**c**) SM index, and (**d**) FM index. SM, skeletal muscle; FM, fat mass. Δ = value at discharge minus value at admission.

To clarify the baseline characteristics of patients with accelerated decrease in body composition, patients were stratified into two groups according to the degree of ΔSM index (value at discharge minus value at admission): those in the lowest quartile of ΔSM index (<−0.4) and the remaining. Patients in the lowest quartile of the ΔSM index showed lower serum total protein levels at admission than the remaining patients (Table 3). Multivariate logistic regression analysis showed that only serum total protein level was independently associated with the lowest quartile of ΔSM index after adjusting for age and sex (Table 4). When the length of hospitalization was included in the analysis, the results did not change. Higher serum CRP levels tended to be associated with the lowest quartile of the ΔSM index. The same was true when the dependent variable was set to the lowest quartile of the ΔFFM index (<−0.7) instead of the ΔSM index. Although there was no significant reduction in FM or FM index during the hospitalization, there were weak negative associations between the length of hospitalization and ΔFM (ρ = −0.31, *p* = 0.01) or ΔFM index (ρ = −0.31, *p* = 0.01) (Figure 3).

## 4. Discussion

To our knowledge, this is the first study to reveal the influence of hospitalization owing to CAP on changes in body composition. Our study had three major findings. First, the body weight and BMI significantly decreased during hospitalization in patients with CAP. Second, SM, SM index, FFM, and FFM index also significantly decreased; however, FM and FM indices did not, indicating that the decrease in BMI could be attributed to SM reduction. Third, an excessive decrease in SM index (the lowest quartile of ΔSM index) was associated with lower serum total protein at admission, whereas a greater decrease in FM and FM index was associated with longer hospitalization.

As hypothesized, hospitalization owing to pneumonia was associated with a significant decrease in body weight, BMI, and SM. This decrease was unrelated to lung comorbidities or the severity of pneumonia. To date, two groups [4,16] have examined the changes in muscle strength during hospitalization in patients with CAP. Martin-Salvador et al. showed a significant reduction in the strength of the hands and quadriceps during hospitalization, which supports the results of our study. They also showed that the reduction in muscle strength was greater in patients aged ≥ 75 years than in those aged < 75 years [16]. Another study of Jose et al. showed a reduction in the strength of the biceps brachii, deltoids, quadriceps, and hamstrings 10 days after hospitalization. Furthermore, they demonstrated that rehabilitation prevented this reduction significantly [4]. Thus, muscle strength decreases during hospitalization for CAP; however, changes in muscle volume have not yet been elucidated. In contrast, this study clarified the changes in body composition during hospitalization. Body composition has recently been assessed to predict the mortality of COVID-19 hospitalized patients, but not to assess changes during hospitalization [17,18,19]. By assessing changes in body composition, we found that a decrease in BMI was attributable to SM reduction in patients with CAP.

In this study, an excess decrease in SM index was associated with lower serum total protein at admission, which seems plausible given that lower serum total protein was observed in sarcopenic conditions [20]. However, the exact mechanisms underlying this association remain unclear. A possible explanation is that lower serum total protein levels at the onset of pneumonia may delay muscle protein turnover and the recovery of muscle mass afterward, as described later.

Other potential mechanisms underlying SM reduction during hospitalization, such as disuse of the extremities, especially the ambulatory muscles [21,22], may be involved. Hand grip strength in the non-dominant hand, but not in the dominant hand, decreased during hospitalization in this study. However, the ΔSM index was not associated with the length of hospitalization. This might be explained by the fact that, in cases of critical illness, loss of muscle mass and strength occurs mainly in the early phase and slowly recovers thereafter [6]. Although the participants with CAP in this study were not admitted to the intensive care unit and body composition was not measured regularly during hospitalization, it is possible that muscle proteins were similarly degraded in the early phase of CAP, resulting in a loss of muscle mass and strength regardless of the length of hospitalization. Systemic inflammation may be involved in the early-phase breakdown of muscle proteins. Pro-inflammatory cytokines, such as IL-6 and IL-10, increase in patients with pneumonia according to disease severity [23]. IL-6 directly induces SM atrophy in healthy rat models [7]. In a mouse model of aspiration pneumonia, the disease led to atrophy of the skeletal, respiratory, and swallowing systems owing to pro-inflammatory cytokines and autophagy [24]. Although insignificant, higher serum CRP levels at admission were associated with an excessive decline in SM index in the multivariate logistic analysis in this study, which may support the hypothesis.

This study highlights the need for nutritional intervention and rehabilitation in patients with CAP. As rehabilitation reduces early mortality in older patients with aspiration pneumonia [25] and improves muscle strength in patients with CAP [4], the effect of a combination of rehabilitation and nutritional therapy aimed at attenuating the early-phase breakdown of muscle proteins in patients with CAP should be investigated further. Verifying the effect of these interventions may help to prevent the recurrence of pneumonia. The relationship between sarcopenia and pneumonia applies not only to older adults but also to patients who have undergone surgery [8,26]. Preoperative sarcopenia increases the risk of postoperative pneumonia as a complication. However, a randomized controlled trial is needed to prove the effectiveness of interventions in preventing sarcopenia after hospitalization for pneumonia.

However, the role of the adipose tissue in patients with CAP remains unclear. In this study, FM at admission was associated with milder pneumonia severity, which seems inconsistent with recent studies on COVID-19 [18,27,28]. In those studies, visceral adipose tissue was associated with worse outcomes. The discrepancy between our findings and those of COVID-19 studies may be explained by the different nature [29] of COVID-19 pneumonia [27] and CAP, and the differing prevalence of obesity. In this study, only 6% of the participants had a BMI > 25 kg/m^2^ at admission. Appropriate fat accumulation, as an indicator of better nutritional status, may be required to prevent the progression of severe pneumonia. Although the direction of causality is unknown, the association between a greater decrease in FM during hospitalization and longer hospitalization duration may support this concept.

This study had certain limitations. First, the number of patients was relatively small, and the severity of pneumonia was mostly mild to moderate because the current BIA method could not be applied to patients with severe CAP who were unable to stand. However, future research is expected to enable the analysis of patients with severe CAP using a new model that can measure body composition while lying down [30]. Second, nutritional interventions and rehabilitation were not standardized in this study. Nonetheless, the current findings are worth reporting in real-world studies. Third, serum markers such as IL-6, which are associated with muscle strength [7,31], were not measured; however, serum total protein is easy to measure and evaluate in clinical practice. Fourth, physical differences between males and females may have affected the results. However, we believe that this effect was minimized by setting changes in the SM index as the dependent variable. Fourth, the median hospital stay of 12 days exceeds the recommended duration of antibiotic use in the recent guideline [32]. This may be due to the advanced age of the patients in the study and the influence of the Japanese insurance system, which allows for long-term hospitalization. Further consideration is needed to determine if this can be generalized to other countries. Finally, it is unclear whether the results of this study are specific to CAP. Further studies comparing these findings with those of hospitalized patients with different conditions are desirable.

## 5. Conclusions

In summary, body weight and BMI significantly decreased during hospitalization in patients with CAP, which was attributed to SM reduction. Patients with low serum total protein levels at admission are at risk of SM index reduction. In contrast, fat volume decreased with prolonged hospitalization. Therefore, strategies to prevent this phenomenon are warranted.

## Figures and Tables

**Table 1:** Baseline characteristics of the participants.

	*n*= 64
Age (years)	76.1 ± 7.2
Male, *n* (%)	50 (78.1)
Height (cm)	161.8 (155.9–167.2)
Length of stay (days)	12.0 (11.0–17.3)
Length of treatment by antibiotics (days)	11.0 (9.0–15.3)
A-DROP (score)	1 (1–2)
A-DROP severity class, *n* (%)	
Mild (0)	9 (14.1)
Moderate (1–2)	49 (76.6)
Severe (3–5)	6 (9.4)
CURB-65 (score)	1 (1–2)
CURB-65 severity class, *n* (%)	
Mild (0–1)	39 (60.9)
Moderate (2)	24 (37.5)
Severe (3–5)	1 (1.6)
Comorbidity, *n* (%)	
Chronic obstructive pulmonary disease	24 (37.5)
Interstitial lung disease	10 (15.6)
Lung cancer (postoperative)	5 (7.8)
Bronchial asthma	3 (4.7)
Non-tuberculous mycobacteria	2 (3.1)
Pulmonary aspergillosis	1 (1.6)
Sarcoidosis	1 (1.6)
Tuberculosis	1 (1.6)

Values are shown as the means ± standard deviations, the number with % in parentheses, or the median with inter-quartile range in parentheses.

**Table 2:** Comparison between variables at admission and at discharge (*n* = 64).

Variables	At Admission	At Discharge	*p* Value
WBC (×10^3^/mm^3^)	11.8 (9.6–14.3)	6.2 (5.2–7.6)	<0.001
Serum CRP (mg/L)	9.3 (5.7–17.7)	0.7 (0.2–1.3)	<0.001
Serum albumin (g/dL)	3.3 (3.0–3.7)	3.2 (3.0–3.5)	<0.01
Serum total protein (g/dL)	6.9 (6.5–7.3)	6.7 (6.2–7.1)	<0.01
ECOG performance status	3 (2–3)	1 (1–2)	<0.001
Weight (kg)	52.9 (46.6–61.7)	52.7 (45.3–61.4)	<0.001
Male	56.0 (48.6–66.1)	54.2 (47.8–62.2)	<0.001
Female	44.8 (40.7–51.5)	44.3 (39.9–50.8)	0.06
BMI (kg/m^2^)	20.5 (18.9–23.0)	20.2 (18.9–23.0)	<0.001
Male	20.6 (19.1–23.9)	20.4 (18.5–23.3)	<0.001
Female	20.2 (18.8–21.8)	19.9 (18.6–21.4)	0.06
SM (kg)	21.4 (18.6–25.3)	20.7 (17.9–25.2)	<0.001
Male	22.9 (19.9–26.5)	22.3 (20.0–26.3)	<0.001
Female	15.3 (14.2–18.9)	15.1 (13.8–18.2)	<0.01
SM index (kg/m^2^)	8.3 (7.3–9.2)	8.1 (7.1–9.0)	<0.001
Male	8.6 (7.8–9.4)	8.5 (7.6–9.2)	<0.001
Female	6.9 (6.3–7.9)	6.7 (6.2–7.5)	0.01
FM (kg)	13.2 (9.0–17.3)	13.2 (9.0–17.3)	0.32
Male	12.8 (8.5–17.9)	11.7 (8.5–18.1)	0.11
Female	15.0 (12.3–16.8)	15.0 (13.3–17.1)	0.39
FM index (kg/m^2^)	5.3 (3.2–6.8)	5.0 (3.3–6.9)	0.35
Male	4.8 (3.1–6.5)	4.4 (3.2–6.5)	0.11
Female	6.7 (6.1–7.1)	6.7 (5.8–7.1)	0.37
FFM (kg)	39.9 (35.4–46.8)	38.9 (34.4–47.1)	<0.001
Male	43.3 (37.8–49.5)	41.5 (37.7–49.3)	<0.01
Female	30.0 (27.9–36.0)	29.7 (27.0–35.1)	0.02
FFM index (kg/m^2^)	15.5 (13.8–17.2)	15.2 (13.9–16.8)	<0.001
Male	15.9 (14.7–17.5)	15.9 (14.2–16.9)	<0.01
Female	13.4 (12.6–15.1)	13.1 (12.4–14.6)	0.02
ECW/TBW	0.396 (0.390–0.403)	0.395 (0.390–0.401)	0.56
Male	0.395 (0.389–0.403)	0.394 (0.389–0.403)	0.40
Female	0.396 (0.393–0.402)	0.398 (0.391–0.402)	0.71
Handgrip strength, dominant hand (kg)	24.0 (19.0–30.3)	25.2 (18.7–30.7)	0.08
Male	26.4 (21.5–31.6)	27.5 (21.6–33.0)	0.32
Female	16.4 (13.1–18.0)	16.7 (14.5–18.4)	0.02
Handgrip strength, non-dominant hand (kg)	22.6 (17.1–28.4)	22.5 (18.1–29.5)	0.01
Male	23.6 (20.2–30.3)	24.1 (19.6–31.0)	0.08
Female	14.9 (12.4–17.1)	16.0 (13.5–19.0)	<0.01
Maximum tongue strength (kPa)	27.8 (21.9–33.6)	28.8 (22.7–35.2)	0.08
FOIS	7 (7–7)	7 (7–7)	0.57

Values are shown as the medians with inter-quartile ranges in parentheses. The numbers of male and female patients are 50 and 14, respectively. CRP, C-reactive protein; WBC, white blood cell; ECOG, Eastern Cooperative Oncology Group; BMI, body mass index; SM, skeletal muscle; FM, fat mass; FFM, fat-free mass; ECW/TBW, extracellular water/total body water; FOIS, food oral intake scales.

**Table 3:** Comparison of variables at admission between patients in the lowest quartile of ΔSM index (<−0.4) and the remaining (*n* = 64).

	ΔSM Index < −0.4 (*n* = 16)	ΔSM Index ≥ −0.4 (*n* = 48)	*p* Value
Weight (kg)	56.9 (52.2–66.3)	50.8 (44.1–61.1)	0.07
Age (years)	74.9 ± 8.1	77.0 ± 6.8	0.46
Sex (male/female)	14/2	36/12	0.30
Length of hospitalization (days)	13.5 (11.0–20.3)	12.0 (11.0–16.3)	0.43
A-DROP (score)	1.5 (1.0–2.0)	1.0 (1.0–2.0)	0.52
CURB-65 (score)	1.0 (1.0–2.0)	1.0 (1.0–2.0)	0.24
CRP (mg/L)	16.5 (8.3–19.7)	8.4 (5.5–15.3)	0.15
Serum albumin (g/dL)	3.3 (3.0–3.6)	3.4 (3.0–3.7)	0.48
Serum total protein (g/dL)	6.6 (6.3–7.0)	7.0 (6.7–7.3)	<0.05
BMI (kg/m^2^)	21.6 (20.4–24.1)	20.4 (18.3–22.9)	0.13
FM index (kg/m^2^)	6.5 (4.4–7.2)	4.8 (3.0–6.8)	0.27

Values are shown as the means ± standard deviations, or the median with inter-quartile range in parentheses. SM, skeletal muscle; CRP, C-reactive protein; BMI, body mass index; FM, fat mass; ΔSM index = SM index value at discharge minus value at admission.

**Table 4:** Multivariate logistic regression analysis for predicting patients with the lowest quartile of the ΔSM index.

Variables	Odds Ratio	95% CI	*p* Value
Age (years)	1.08	0.98–1.19	0.14
Sex (female)	1.70	0.25–11.46	0.58
Serum total protein at admission (g/dL)	7.88	1.70–36.62	0.008
Weight at admission (kg)	0.95	0.90–1.01	0.11

SM, skeletal muscle; ΔSM index = SM index value at discharge minus value at admission.

## Data Availability

The datasets used and/or analyzed during the current study are available from the corresponding author on reasonable request.

## Abbreviations

The following abbreviations are used in this manuscript:

BIA	bioelectrical impedance analysis
BMI	body mass index
CAP	community-acquired pneumonia
CRP	C-reactive protein
ECOG	Eastern Cooperative Oncology Group Performance Status Scale
FFM	fat-free mass
FM	fat mass
SM	skeletal muscle

## References

[1] Huang S., Guo Y., Chen L., Wang Y., Chen X. (2022). Clinical muscle mass-related biomarkers that predict mortality in older patients with community-acquired pneumonia. BMC Geriatr..

[2] Bylova N.A., Arutyunov G.P., Rylova A.K., Simbirtseva A.S., Arutyunov A.G. (2017). Prognostic role of body composition in patients with pneumonia associated with decompensated CHF. Kardiologiia.

[3] The Committee for the Japanese Respiratory Society (2017). Guidelines in the management of respiratory infections. The JRS Guidelines for the Management of Pneumonia in Adults.

[4] José A., Dal Corso S. (2016). Inpatient rehabilitation improves functional capacity, peripheral muscle strength and quality of life in patients with community-acquired pneumonia: A randomised trial. J. Physiother..

[5] Martín-Salvador A., Colodro-Amores G., Torres-Sánchez I., Moreno-Ramírez M.P., Cabrera-Martos I., Valenza M.C. (2016). Physical therapy intervention during hospitalization in patients with acute exacerbation of chronic obstructive pulmonary disease and pneumonia: A randomized clinical trial. Med. Clin..

[6] van Gassel R.J.J., Baggerman M.R., van de Poll M.C.G. (2020). Metabolic aspects of muscle wasting during critical illness. Curr. Opin. Clin. Nutr. Metab. Care.

[7] Haddad F., Zaldivar F., Cooper D.M., Adams G.R. (2005). IL-6-induced skeletal muscle atrophy. J. Appl. Physiol..

[8] Okazaki T., Ebihara S., Mori T., Izumi S., Ebihara T. (2020). Association between sarcopenia and pneumonia in older people. Geriatr. Gerontol. Int..

[9] Miyashita N., Matsushima T., Oka M., Japanese Respiratory Society (2006). The JRS guidelines for the management of community-acquired pneumonia in adults: An update and new recommendations. Intern. Med..

[10] Lim W.S., van der Eerden M.M., Laing R., Boersma W.G., Karalus N., Town G.I., Lewis S.A., Macfarlane J.T. (2003). Defining community acquired pneumonia severity on presentation to hospital: An international derivation and validation study. Thorax.

[11] Oken M.M., Creech R.H., Tormey D.C., Horton J., Davis T.E., McFadden E.T., Carbone P.P. (1982). Toxicity and response criteria of the Eastern Cooperative Oncology Group. Am. J. Clin. Oncol..

[12] Crary M.A., Mann G.D., Groher M.E. (2005). Initial psychometric assessment of a functional oral intake scale for dysphagia in stroke patients. Arch. Phys. Med. Rehabil..

[13] Nishiyama O., Yamazaki R., Sano H., Iwanaga T., Higashimoto Y., Kume H., Tohda Y. (2017). Fat-free mass index predicts survival in patients with idiopathic pulmonary fibrosis. Respirology.

[14] Rinaldi S., Gilliland J., O’Connor C., Seabrook J.A., Mura M., Madill J. (2021). Fat-free mass index controlled for age and sex and malnutrition are predictors of survival in interstitial lung disease. Respiration.

[15] Takahashi M., Koide K., Suzuki H., Satoh Y., Iwasaki S.I. (2016). Evaluation of reliability of perioral muscle pressure measurements using a newly developed device with a lip piece. Acta Bioeng. Biomech..

[16] Martín-Salvador A., Torres-Sánchez I., Sáez-Roca G., López-Torres I., Rodríguez-Alzueta E., Valenza M.C. (2015). Age group analysis of psychological, physical and functional deterioration in patients hospitalized for pneumonia. Arch. Bronconeumol..

[17] Hosch R., Kattner S., Berger M.M., Brenner T., Haubold J., Kleesiek J., Koitka S., Kroll L., Kureishi A., Flaschel N. (2022). Biomarkers extracted by fully automated body composition analysis from chest CT correlate with SARS-CoV-2 outcome severity. Sci. Rep..

[18] Graziano E., Peghin M., De Martino M., De Carlo C., Da Porto A., Bulfone L., Casarsa V., Sozio E., Fabris M., Cifu A. (2022). The impact of body composition on mortality of COVID-19 hospitalized patients: A prospective study on abdominal fat, obesity paradox and sarcopenia. Clin. Nutr. ESPEN.

[19] Besutti G., Pellegrini M., Ottone M., Bonelli E., Monelli F., Fari R., Milic J., Dolci G., Fasano T., Canovi S. (2022). Modifications of chest CT body composition parameters at three and six months after severe COVID-19 pneumonia: A retrospective cohort study. Nutrients.

[20] Sanad T., Ali S.H., Alsadany M.A., EI Banouby M.H. (2019). Prevalence of sarcopenia among hospitalized elderly patients. Egypt. J. Geriatr. Gerontol..

[21] Pitta F., Troosters T., Probst V.S., Spruit M.A., Decramer M., Gosselink R. (2006). Physical activity and hospitalization for exacerbation of COPD. Chest.

[22] Burtin C., Van Remoortel H., Vrijsen B., Langer D., Colpaert K., Gosselink R., Decramer M., Dupont L., Troosters T. (2013). Impact of exacerbations of cystic fibrosis on muscle strength. Respir. Res..

[23] Martínez R., Menéndez R., Reyes S., Polverino E., Cillóniz C., Martínez A., Esquinas C., Filella X., Ramírez P., Torres A. (2011). Factors associated with inflammatory cytokine patterns in community-acquired pneumonia. Eur. Respir. J..

[24] Komatsu R., Okazaki T., Ebihara S., Kobayashi M., Tsukita Y., Nihei M., Sugiura H., Niu K., Ebihara T., Ichinose M. (2018). Aspiration pneumonia induces muscle atrophy in the respiratory, skeletal, and swallowing systems. J. Sarcopenia Muscle.

[25] Momosaki R., Yasunaga H., Matsui H., Horiguchi H., Fushimi K., Abo M. (2015). Effect of early rehabilitation by physical therapists on in-hospital mortality after aspiration pneumonia in the elderly. Arch. Phys. Med. Rehabil..

[26] Shu X., Song Q., Huang X., Tang T., Huang L., Zhao Y., Lin T., Xu P., Yu P., Yue J. (2025). Sarcopenia and risk of postoperative pneumonia: A systematic review and meta-analysis. J. Nutr. Health Aging.

[27] Surov A., Thormann M., Kardas H., Hinnerichs M., Omari J., Cingöz E., Cingöz M., Dursun M., Kormaz İ., Orhan Ç. (2023). Visceral to subcutaneous fat ratio predicts short-term mortality in patients with Covid 19. A multicenter study. Br. J. Radiol..

[28] Bunnell K.M., Thaweethai T., Buckless C., Shinnick D.J., Torriani M., Foulkes A.S., Bredella M.A. (2021). Body composition predictors of outcome in patients with COVID-19. Int. J. Obes..

[29] Steenblock C., Schwarz P.E.H., Ludwig B., Linkermann A., Zimmet P., Kulebyakin K., Tkachuk V.A., Markov A.G., Lehnert H., de Angelis M.H. (2021). COVID-19 and metabolic disease: Mechanisms and clinical management. Lancet Diabetes Endocrinol..

[30] Darroch P., O’Brien W.J., Mazahery H., Wham C. (2022). Sarcopenia prevalence and risk factors among residents in aged care. Nutrients.

[31] Spruit M.A., Gosselink R., Troosters T., Kasran A., Gayan-Ramirez G., Bogaerts P., Bouillon R., Decramer M. (2003). Muscle force during an acute exacerbation in hospitalised patients with COPD and its relationship with CXCL8 and IGF-I. Thorax.

[32] Jones B.E., Ramirez J.A., Oren E., Soni N.J., Sullivan L.R., Restrepo M.I., Musher D.M., Erstad B.L., Pickens C., Vaughn V.M. (2025). Diagnosis and Management of Community-acquired Pneumonia. An Official American Thoracic Society Clinical Practice Guideline. Am. J. Respir. Crit. Care Med..

